# Comparative proteomics analysis of biofilms and planktonic cells of *Enterococcus faecalis* and *Staphylococcus lugdunensis* with contrasting biofilm-forming ability

**DOI:** 10.1371/journal.pone.0298283

**Published:** 2024-05-29

**Authors:** Jung-Ah Cho, Sangsoo Jeon, Youngmin Kwon, Yoo Jin Roh, Chang-Hun Lee, Sung Jae Kim

**Affiliations:** 1 Department of Orthopedic Surgery, Dongtan Sacred Hospital, Hallym University, Hwaseong, Republic of Korea; 2 College of Transdisciplinary Studies, School of Undergraduate Studies, Daegu Gyeongbuk Institute of Science and Technology, Daegu, Republic of Korea; 3 Department of New Biology, Daegu Gyeongbuk Institute of Science and Technology, Daegu, Republic of Korea; BOKU: Universitat fur Bodenkultur Wien, AUSTRIA

## Abstract

Biofilms make it difficult to eradicate bacterial infections through antibiotic treatments and lead to numerous complications. Previously, two periprosthetic infection-related pathogens, *Enterococcus faecalis* and *Staphylococcus lugdunensis* were reported to have relatively contrasting biofilm-forming abilities. In this study, we examined the proteomics of the two microorganisms’ biofilms using LC-MS/MS. The results showed that each microbe exhibited an overall different profile for differential gene expressions between biofilm and planktonic cells as well as between each other. Of a total of 929 proteins identified in the biofilms of *E*. *faecalis*, 870 proteins were shared in biofilm and planktonic cells, and 59 proteins were found only in the biofilm. In *S*. *lugdunensis*, a total of 1125 proteins were identified, of which 1072 proteins were found in common in the biofilm and planktonic cells, and 53 proteins were present only in the biofilms. The functional analysis for the proteins identified only in the biofilms using UniProt keywords demonstrated that they were mostly assigned to membrane, transmembrane, and transmembrane helix in both microorganisms, while hydrolase and transferase were found only in *E*. *faecalis*. Protein-protein interaction analysis using STRING-db indicated that the resulting networks did not have significantly more interactions than expected. GO term analysis exhibited that the highest number of proteins were assigned to cellular process, catalytic activity, and cellular anatomical entity. KEGG pathway analysis revealed that microbial metabolism in diverse environments was notable for both microorganisms. Taken together, proteomics data discovered in this study present a unique set of biofilm-embedded proteins of each microorganism, providing useful information for diagnostic purposes and the establishment of appropriately tailored treatment strategies. Furthermore, this study has significance in discovering the target candidate molecules to control the biofilm-associated infections of *E*. *faecalis* and *S*. *lugdunensis*.

## Introduction

Bacterial biofilms are well-organized microbial communities that are embedded in self-produced extracellular polymeric substances (EPS) including polysaccharides, proteins, and lipids [1]. The overall processes of biofilm formation involve the following steps: (1) planktonic cells adhere and colonize to a surface, (2) microcolonies and matrices are formed, (3) biofilms are matured and then dispersed [2]. Until now, various biological mechanisms including quorum sensing, outer membrane structure, stress responses, etc. were known to regulate biofilm formation [3]. Once formed, biofilm becomes highly resistant to antibiotic treatments for bacterial infection removal, and thus leads to numerous complications [4–8]. Furthermore, biofilm can form on surfaces of water pipe walls [9] as well as surgical implants and catheters [10], and so the issue related to biofilm formation is regarded as an important problem to be solved not only in the medical field but also in everyday life.

To overcome the challenges posed by biofilms, a better understanding of biofilm formation in diverse microorganisms is essential. In particular, we believed that it is important to have a comprehensive understanding of the biofilm-related behaviors of medically relevant pathogens because they are closely related to our health. For the reasons, in our previous studies, we analyzed whether the biofilm-forming ability of a specific microorganism changed when a surface structure was structurally modified [11], and assessed the biofilm-forming abilities of a broad spectrum of periprosthetic infection-associated pathogens on the surfaces frequently provided in medical fields [12]. The studies demonstrated that the biofilm-forming ability of a certain microorganism could vary depending on the material and structure of a given surface, and not all pathogens that are more frequently found in biofilm-associated infectious situations were highly biofilm-forming. Through our previous works, we drew attention to two microbes, *Staphylococcus lugdunensis* and *Enterococcus faecalis*, which showed opposite trends in the degree of biofilm formation on the test surfaces; *S*. *lugdunensis* had the highest biofilm-forming ability and *E*. *faecalis* had the lowest. Although *Staphylococcus aureus* and *Pseudomonas aeruginosa* are the two representative bacteria species identified as major causes of serious infectious diseases [13], coagulase-negative *staphylococci* such as *Staphylococcus lugdunensis* are also known to cause many periprosthetic joint infections [14], and *Enterococcus* species are also reported as noteworthy pathogenic bacteria associated with medical implants [15].

From our previous study [12], we became curious about how the biofilms of each microbe differed biologically from the cells in planktonic growth mode, and how their biofilms differed from each other. As biofilm is the result of a microbial response through changes in gene expression in response to internal or external stimuli, we believed that the unique characteristics of biofilm formation could be figured out by analyzing proteins.

To investigate the major proteomic factors that distinguished the difference in the biofilm-forming abilities, proteomics using LC-MS/MS was performed on the biofilms and planktonic cells of the two microbes, and analyzed with bioinformatics tools. The results showed that proteins belonging to hydrolase such as guanine deaminase and transport/transferase including phosphotransferase system (PTS) were notably found in the weak biofilm-forming *E*. *faecalis*, compared to the strong biofilm-forming *S*. *lugdunensis*. The results indicate that those proteins can be utilized for diagnostic purposes and as an initial approaching point in establishing a strategy in order to properly control biofilm formation.

## Materials and methods

### Bacteria culture

*Enterococcus faecalis* and *Staphylococcus lugdunensis* were obtained from the National Culture Collection for Pathogens (NCCP, Korea), which was established as the national pathogen resources bank in order to the promotion of R&D in preparedness, diagnosis, and therapy of infectious human disease. *Enterococcus faecalis* (NCCP 15611) or *Staphylococcus lugdunensis* (NCCP 15630) are originally isolated from pus or blood, respectively. Tryptic soy agar (TSA, Difco) or tryptic soy broth (TSB, Difco) was used to culture the bacteria unless specified. A primary bacterial culture was prepared by inoculating one single colony on agar plates into broth media and incubating overnight at 37°C with shaking at 120 rpm.

### Biofilm formation

All experimental procedures or materials, devices, and equipment were aseptically performed or maintained; a lack of cross-contamination was confirmed using empty plates. The primary culture was diluted with fresh broth media to achieve an optical density (OD) at 600 nm (OD600) value of 0.9 to 1.0 (DeNovix DS-C Spectrophotometer) and then, 1 mL of the diluted bacterial suspension was dispensed into 2~5 of 14-mL round-bottom tubes (40114; SPL Life Sciences, Korea) that was sterilized by gamma irradiation, followed by incubation at 37°C with shaking at 50 rpm. After 72 h, the non-adherent planktonic cells and the culture tubes were separately collected. Planktonic cells were harvested by centrifugation and then lysed in RIPA buffer to obtain protein extract. The culture tubes were added with glass beads of 2 mm diameter and vortexed to detach biofilm formed on the tube surface, and the process was repeated 3 times. The amount of extracted protein was determined by BCA assay. The protein sample preparation for proteomics was conducted independently three times.

### LC-MS/MS proteomics

The quantified protein samples were delivered to a proteomics service company and proceeded for proteome analysis by following the procedures; FASP digestion, desalting, and LS-MS/MS analysis. Briefly, protein samples were reduced by incubation with 5 mM TCEP at 37°C for 30 min and then alkylated with 50 mM IAA in the dark at 25°C for 1 h, followed by adding 8M urea for 15 min. After that, trypsin in 50 mM ABC was added and incubated at 37°C for 18 h, followed by stopping the reaction by adding formic acid (pH 2). Desalting was carried out with a C18 micro spin column that had been prepared with 100% methanol, 0.1% formic acid, and 80% ACN, followed by drying with speed-vac. The samples were stored at –20°C until analysis. Finally, the samples were subjected to LC-MS/MS analysis using UPLC/Q-Exactive. The parameters and conditions for LC-MS/MS analysis are as follows;

C18, 3 μm, 100 Å, 75 μm x 2 cm for trapping column; PepMapTM RSLC C18, 2 μm, 100 Å, 75 μm x 50 cm for analytical column; Water with 0.1% formic acid (A) and 80% ACN with 0.1% formic acid (B) for mobile phase; 0, 14, 120, 120.1, 130, 130.1, 180 min (time) and 4, 4, 40, 96, 96, 4, 4% (solvent B) for gradient; 300 nL/min for column flow rate; 400~2000 m/z for mass range. The peptides of each sample isolated by LS-MS (S1 Fig) were identified by Proteome discoverer using Uniprot *Enterococcus faecalis* or *Staphylococcus lugdunensis* databases (https://www.uniprot.org). Abundances were normalized based on BCA protein assay. Experiments were repeated twice, and each experimental group contained 3 independently prepared samples (n = 2–5).

### Data analysis and processing

Among all identified peptides or proteins, only those identified in common from the repeated experiments were selected with a false discovery rate (FDR) at 1%, and overlapping items or uncharacterized proteins were excluded from analyses. Also, the same protein for different gene names was unified. The selected protein lists were organized based on gene names. Data analysis and processing displayed as Venn diagram, cluster heatmap, gene ontology (GO) enrichment analysis, scatter plot, etc. was primarily performed using the following web-based tools; https://www.bioinformatics.com.cn/srplot and http://bioinformatics.sdstate.edu/go/. For GO, protein-protein network, and KEGG pathway analysis, the following web was also used; https://string-db.org. For statistical analysis between protein abundances of biofilm and planktonic cell group, unpaired or paired t-tests were performed and P<0.05 was considered to indicate a statistically significant difference.

## Results

### Identification of proteins of biofilm and planktonic cells

**Fig 1:**
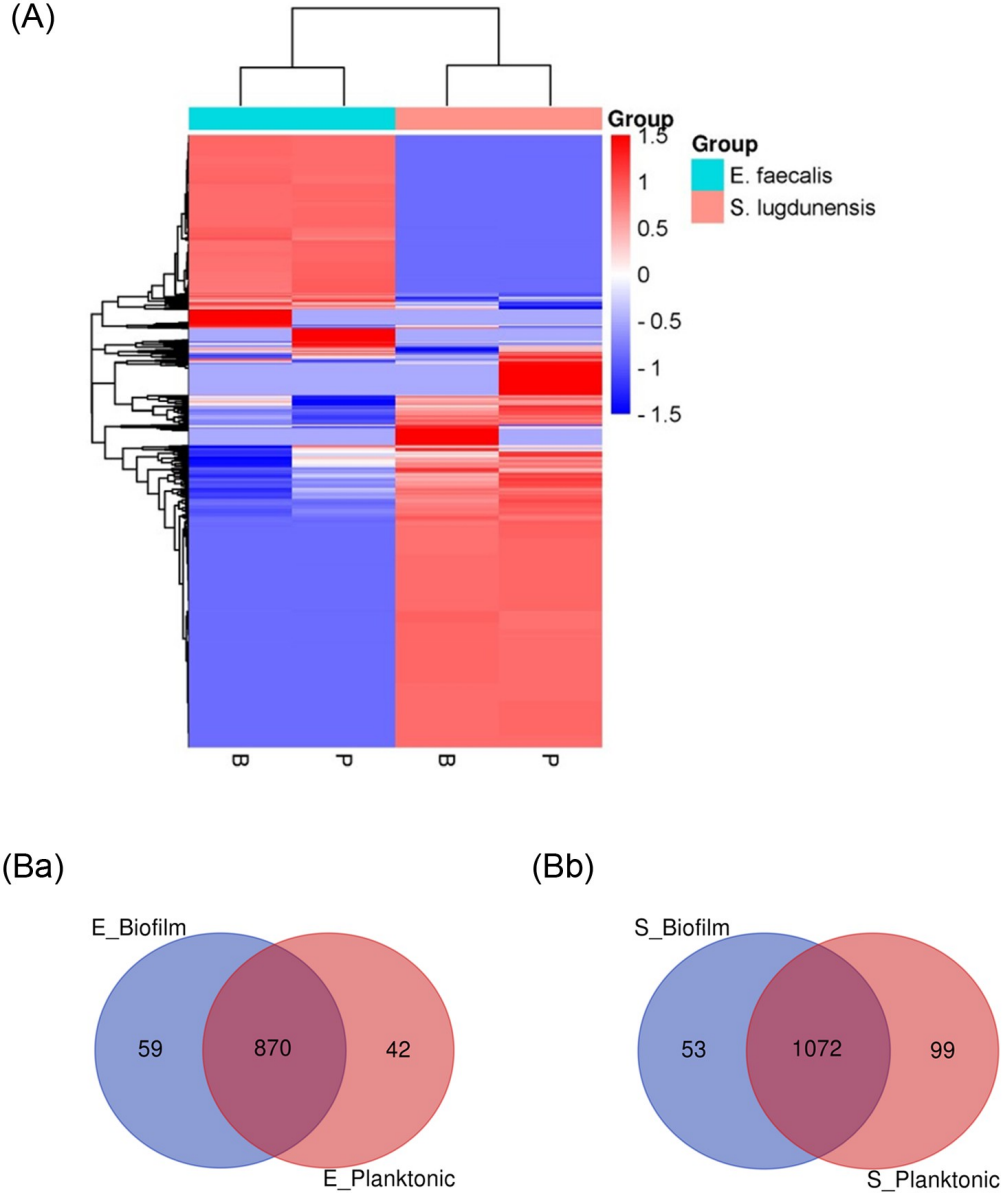
A. Clustered heat map for the relative abundance of proteins identified in the indicated sample. All the proteins in biofilms (B) and planktonic cells (P) of *E*. *faecalis* or *S*. *lugdunensis* were aligned and relatively compared at the abundance basis. The color scale [blue (lower levels) to red (higher levels)] represents the abundance of each protein across different samples. Fig 1B. Venn diagrams for the proteins identified in biofilm and planktonic cells of *E*. *faecalis* (a) and *S*. *lugdunensis* (b). **a.** For *E*. *faecalis*, a total of 971 proteins were identified in biofilm and planktonic cells, of which 870 were in common, and 59 or 42 were found only biofilm or planktonic cells, respectively. **b**. in *S*. *lugdunensis*, a total of 1224 proteins were identified in biofilm and planktonic cells, of which 1072 were in common, and 53 or 99 were found only in biofilm or planktonic cells, respectively.

The protein isolates of biofilm and planktonic cells of *E*. *faecalis* and *S*. *lugdunensis* were subjected to proteomics analysis using LC-MS. As a result, a total of 971 or 1224 proteins were identified in biofilms and planktonic cells of *E*. *faecalis* (929 and 912 proteins) or *S*. *lugdunensis* (1125 and 1171 proteins), respectively (S1 and S2 Tables). To intuitively understand the protein composition of the biofilm and planktonic cell of the two microbes, the genes, and abundances of the proteins identified in the two microbes were subjected together to the clustering heatmap (Fig 1A). The result revealed completely different expression patterns between *E*. *faecalis* and *S*. *lugdunensis*. As expected, the protein enrichment patterns were found to be markedly distinguishable, not only between the biofilm and planktonic cells of each microbe, but also between the biofilms of the two microbes. The proteins identified in each microorganism were then analyzed separately.

Then, the proteins identified in each microbe were individually analyzed. In the case of *E*. *faecalis*, 929 proteins in biofilms and 912 proteins in planktonic cells were identified, among which 870 proteins were found in common, 59 proteins were only in biofilm, and 42 proteins were only in planktonic cells (Fig 1B and 1a). On the other hand, in the case of *S*. *lugdunensis*, a total of 1125 proteins or 1171 proteins were identified in biofilms or planktonic cells, respectively, among which 1072 proteins were shared in common, 53 proteins were identified only in biofilm, and 99 proteins existed only in planktonic cells (Fig 1B and 1b).

### Analysis of the commonly-identified proteins based on abundance level

**Fig 2:**
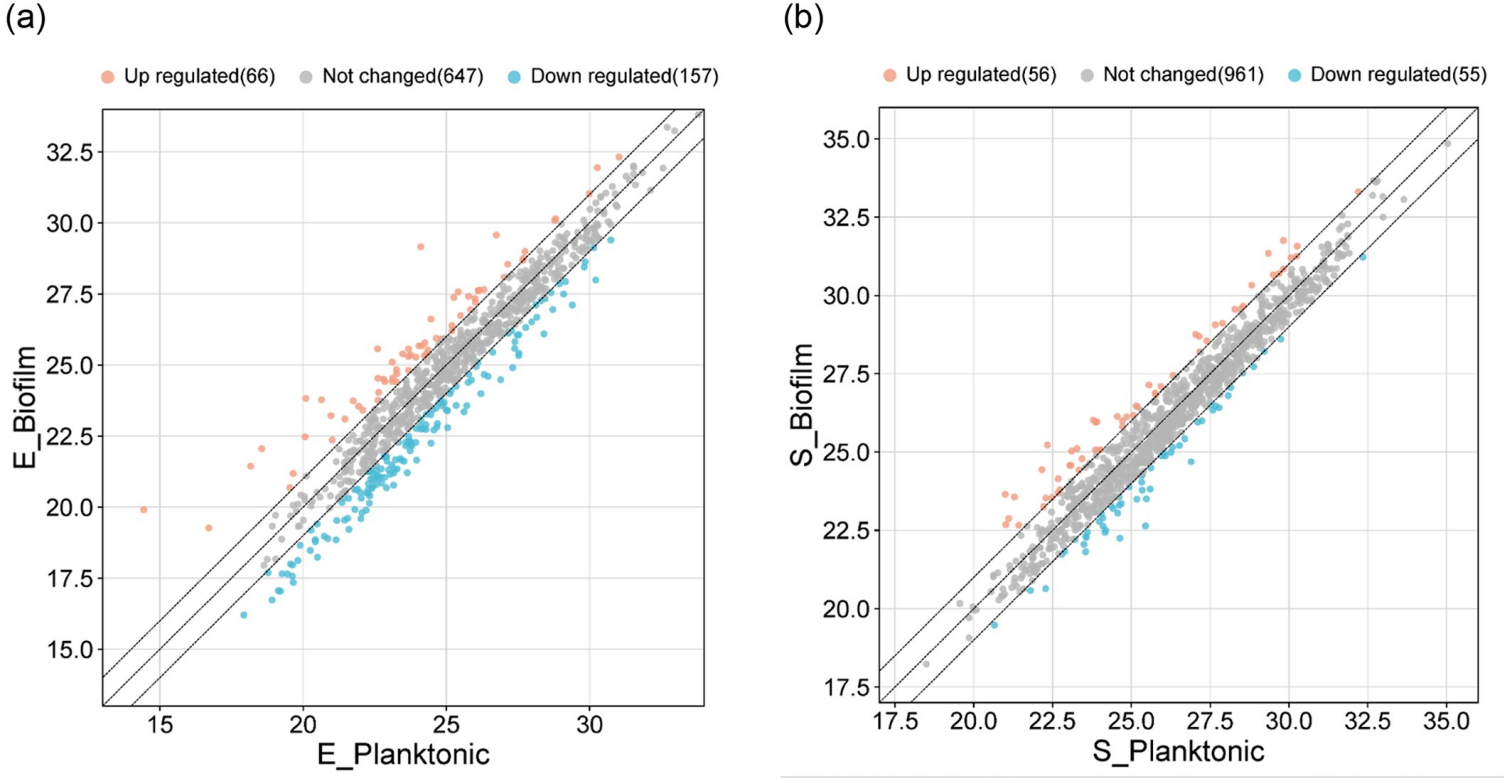
Scatter plots for the proteins identified in common in biofilm and planktonic cells of *E*. *faecalis* (A) and *S*. *lugdunensis* (B). **A.** Of a total of 870 proteins of *E*. *faecalis*, 66 or 157 proteins were relatively more abundant in biofilms or planktonic cells, respectively, and 647 proteins were at similar levels. **B.** Of a total of 1072 proteins of *S*. *lugdunensis*, 56 or 55 proteins were relatively more abundant in biofilms or planktonic cells, respectively, and 961 proteins were at similar levels. The x- or y-axis scales represent log2 values of the protein abundances from each indicated group, where the fold change cutoff is 2.

The proteins identified in common in both biofilm and planktonic cells of each microbe were further analyzed for the expression level (Fig 2). Among the 870 proteins of *E*. *faecalis*, 66 proteins appeared to be more abundant in biofilm than in planktonic cells, and 157 proteins were less abundant, while 647 proteins showed no significant difference between the groups (Fig 2A). In the case of *S*. *lugdunensis*, 56 proteins out of 1072 proteins were found to be more abundant in biofilm than in planktonic cells, and the other 55 proteins were less abundant, while 961 proteins were in the range of no change (Fig 2B).

The proteins commonly identified in both biofilms and planktonic cells were calculated for up- or down-regulation in biofilms by dividing the abundance in biofilm by that in planktonic cells (B/P). When ordered by B/P ratios, the 10 proteins with the highest or lowest B/P ratios are shown in Table 1.

**Table 1:** A list of the top 10 proteins that are relatively more abundant in either biofilm (*white panel*) or planktonic cell (*gray panel*) among those commonly identified in biofilms and planktonic cells of *E*. *faecalis* or *S*. *lugdunensis*.

ID	Accession	Gene name	Description	Abundance [log2]		Ratio
				Biofilm	Planktonic cell	(B/P)
*E*. *faecalis*	Q830L4	recR	Recombination protein RecR	19.91	14.43	1.38
	H7C6X6	EF_0394	Secreted antigen, putative	29.16	24.11	1.21
	Q833B5	EF_2049	ABC transporter, permease protein, putative	22.05	18.56	1.19
	Q832N0	rfbC	dTDP-4-dehydrorhamnose 3,5-epimerase	23.82	20.09	1.19
	Q836T6	EF_1021	N-acetyltransferase domain-containing protein	21.44	18.17	1.18
	Q830T2	sbcD	Nuclease SbcCD subunit D	19.26	16.71	1.15
	Q835S3	EF_1302	Transcriptional regulator, putative	23.78	20.65	1.15
	Q834U5	xerD	Tyrosine recombinase XerD	25.57	22.60	1.13
	Q831Q4	EF_2446	DNA_pol3_delta domain-containing protein	22.48	20.07	1.12
	Q831K0	EF_1093	Cell wall surface anchor family protein	23.22	20.97	1.11
	Q838Z3	EF_0289	Cysteine synthase B, putative	19.89	22.20	0.90
	Q837D9	mvk	Mevalonate kinase	17.57	19.62	0.90
	Q830V6	recD2	ATP-dependent RecD-like DNA helicase	17.06	19.14	0.89
	H7C6X2	pheA	Prephenate dehydratase	18.85	21.15	0.89
	Q830J1	EF_2791	5-formyltetrahydrofolate cyclo-ligase	19.59	22.02	0.89
	Q839V4	mrnC	Mini-ribonuclease 3	18.24	20.50	0.89
	Q839A9	EF_3207	tRNA-dihydrouridine synthase	19.22	21.65	0.89
	Q832Z3	EF_0180	ABC transporter, permease protein	17.04	19.20	0.89
	Q835M8	EF_1349	Alpha-amylase	16.73	18.92	0.88
	Q835M9	EF_1348	Glucan 1,6-alpha-glucosidase, putative	17.35	19.66	0.88
*S*. *lugdunensis*	A0A292DIE8	EQ812_06280	Cold shock protein CspA	25.22	22.34	1.13
	A0A133Q308	EQ812_07575	Tautomerase	23.65	21.00	1.13
	A0A292DIK1	metE	5-methyltetrahydropteroyltriglutamate—homocysteine S-methyltransferase	23.57	21.29	1.11
	A0A4Q9WCW5	tkt	Transketolase	24.44	22.16	1.10
	A0A133Q357	dapB	4-hydroxy-tetrahydrodipicolinate reductase	26.02	23.79	1.09
	A0A292DJ14	budA	Alpha-acetolactate decarboxylase	25.96	23.87	1.09
	A0A4Q9WED6	EQ812_01705	DUF2188 domain-containing protein	25.97	23.91	1.09
	A0A4Q9WB32	EQ812_01820	YSIRK signal domain/LPXTG anchor domain surface protein	22.88	21.11	1.08
	A0A4Q9W829	EQ812_12000	TatD family deoxyribonuclease	25.03	23.10	1.08
	A0A4Q9W019	EQ812_14855	AAA family ATPase (Fragment)	22.68	21.02	1.08
	A0A133Q3R6	miaA	tRNA dimethylallyltransferase	23.50	25.18	0.93
	A0A292DH81	glpT	Glycerol-3-phosphate transporter	22.51	24.18	0.93
	A0A133Q2B4	HMPREF3225_01965	Biotin-dependent carboxylase domain protein	23.82	25.59	0.93
	A0A4V2KW16	EQ812_00015	DHA2 family efflux MFS transporter permease subunit	22.43	24.17	0.93
	A0A133Q5C5	EQ812_01190	HTH-type transcriptional regulator SarZ	20.64	22.28	0.93
	A0A133QAB1	rimM	Ribosome maturation factor RimM	21.82	23.55	0.93
	A0A4Q9WBJ7	trhO	tRNA uridine(34) hydroxylase	23.51	25.48	0.92
	A0A4Q9W9W2	EQ812_10645	Acetyl-CoA carboxylase biotin carboxyl carrier protein subunit	24.69	26.90	0.92
	A0A4Q9WB61	cca	CCA-adding enzyme	22.25	24.63	0.90
	A0A4Q9WBQ6	rlmN	Probable dual-specificity RNA methyltransferase RlmN	22.64	25.45	0.89

For *E*. *faecalis*, proteins with the higher ratio of abundance in biofilms relative to abundance in planktonic cells (ratio B/P) include recombination protein RecR (*recR*), secreted antigen, putative (*EF_0394*) and ABC transporter and permease protein, putative (*EF_2049*), while glucan 1,6-alpha-glucosidase, putative (*EF_1348*), Alpha-amylase (*EF_1349*) and ABC transporter, permease protein (*EF_0180*) were found to be more abundant in planktonic cell compared to in biofilm. Aside from the ABC transporter/permease proteins, the finding that recombination protein and secreted antigen were upregulated in the biofilm indicated the dynamics and pathogenicity in the biofilm of the microbe. On the other hand, the finding that glucan 1,6-alpha-glucosidase, putative, and alpha-amylase were upregulated in planktonic cells reflected the more static and fundamental properties of planktonic cells.

In the case of *S*. *lugdunensis*, cold shock protein CspA (*EQ812_06280*), tautomerase (*EQ812_07575*), 5-methyltetrahydropteroyltriglutamate—homocysteine S-methyltransferase (*metE*), etc. were revealed to be more abundant in biofilm than in planktonic cells, while the levels of probable dual-specificity RNA methyltransferase RlmN (*rlmN*), CCA-adding enzyme (*cca*), acetyl-CoA carboxylase biotin carboxyl carrier protein subunit (*EQ812_10645*), etc. appeared to be relatively higher in planktonic cells compared to biofilms. This result demonstrated the sensitive and quick responsiveness of the microorganism to environmental changes in the biofilm through protein modification rather than protein synthesis.

When considering the characteristics of up- or down-regulated proteins in the biofilms of the two microorganisms, there were generally no consistent properties, as expected from their opposing biofilm-forming abilities, implying that the two microorganisms used distinct biological systems for biofilm formation. As predicted from the ratios of abundance in biofilm to abundance in planktonic cell (ratio B/P) ranging from 0.88 to 1.38 or 0.92 to 1.13 for *E*. *faecalis* or *S*. *lugdunensis*, respectively (Table 1), the difference between the protein abundances of biofilm and planktonic cell groups was considered statistically insignificant (p = 0.0857), and thus the proteins identified in common in both biofilms and planktonic cells were excluded from further analysis.

### Comparison of the proteins identified only in either biofilms or planktonic cells

The proteins identified only in either biofilms or planktonic cells were listed up to the top 10 by abundance in Table 2 or S3 Table, respectively. In the case of *E*. *faecalis*, cobalamin synthesis protein/P47K family protein (*EF_3204*), minor structural protein (*EF_2001*), regulatory protein VanRB (*vanRB*), D-alanyl carrier protein (*dltC*), etc., were found to be most abundant in the biofilm, whereas proteins including glycine betaine/L-proline ABC transporter, glycine betaine/L-proline-binding/permease protein (*EF_2642*), o-succinylbenzoate synthase (*menC*), and helix-turn-helix protein, iron-dependent repressor family (*EF_0578*) were found to be most abundant in planktonic cells. Among the most abundant proteins found only in the biofilm, the presence of regulatory protein VanRB and D-alanyl carrier protein, which are involved in antibiotic resistance and endotoxin synthesis, mirrored the virulence of biofilms, which was in contrast to planktonic cell-specific proteins with metabolite transport or synthesis activities.

**Table 2:** A list of the top 10 proteins identified only in biofilms which were not found in planktonic cells.

ID	Accession	Gene name	Description	Abundance [log2]
*E*. *faecalis*	Q82Z69	EF_3204	Cobalamin synthesis protein/P47K family protein	25.42
	Q833F8	EF_2001	Minor structural protein	24.82
	Q47744	vanRB	Regulatory protein VanRB	24.67
	Q830N2	dltC	D-alanyl carrier protein	23.71
	H7C6V1	EF_0500	AAA domain-containing protein	23.36
	Q82ZD2	EF_3132	PC4 domain-containing protein	23.14
	Q838L4	EF_0432	Transcriptional regulator, AraC family	23.09
	Q831E2	EF_2569	NTP_transf_3 domain-containing protein	22.57
	Q836Z3	EF_0950	tRNA threonylcarbamoyladenosine biosynthesis protein TsaE	22.50
	Q839T6	ruvA	Holliday junction ATP-dependent DNA helicase RuvA	22.46
*S*. *lugdunensis*	A0A4Q9WCN8	EQ812_04065	DUF5084 domain-containing protein	26.99
	A0A133Q347	HMPREF3225_01770	ATPase family protein	25.42
	A0A292DDN9	EQ812_09565	Methyltransferase domain-containing protein	24.79
	A0A133Q7S4	HMPREF3225_01013	Putative stage 0 sporulation protein J	24.79
	A0A133QCC6	HMPREF3225_00114	Probable heme-iron transport system permease protein IsdF	24.77
	A0A133Q5X1	HMPREF3225_01307	TIGR00245 family protein	24.64
	A0A133Q7R7	HMPREF3225_00968	Hydratase/decarboxylase	24.23
	A0A133Q3A3	HMPREF3225_01812	THUMP domain protein	23.88
	A0A133QAF3	EQ812_09000	Putative hemin transport system permease protein HrtB	23.88
	A0A4Q9W1N1	EQ812_13690	Thiamine diphosphokinase (Fragment)	23.66

In the case of *S*. *lugdunensis*, DUF5084 domain-containing protein (*EQ812_04065*), ATPase family protein (*HMPREF3225_01770*), Methyltransferase domain-containing protein (*EQ812_09565*), putative stage 0 sporulation protein J (*HMPREF3225_01013*), etc., were abundantly present in the biofilm, while cassette chromosome recombinase A4-like protein (*ccrA4*), LysR substrate binding domain protein (*HMPREF3225_01218*), MoxR family ATPase (*EQ812_07745*), class I SAM-dependent RNA methyltransferase etc., were rich in planktonic cells. Except for putative stage 0 sporulation protein J, whose function has been determined, it was difficult to describe the characteristics of the biofilm of the microorganism through the biofilm-specific proteins at this time. Taken together, we found that the biofilm-specific protein profiles of the two microorganisms exhibited non-identical properties, which suggests that the biofilms of each microorganism may exert biologically different characteristics.

Validation of the proteomics results was performed via RT-qPCR using RNA extracted from the biofilms (S2 Fig). Among the genes found to be abundant in the biofilms, the experiments were conducted on 3 to 4 genes that were available for designing primers and the 16s rRNA gene as an internal control (S4 Table). Cq values for the mRNA expression of each gene obtained from the RT-qPCR (S2a Fig) were subjected to correlation analysis with proteomics data (S2b Fig). The result revealed that the Cq values of the tested genes were inversely related to their abundance levels in the proteomics data, indicating that the differentially expressed proteins from the proteomic analysis were well validated.

### Functional analysis of the proteins identified only in biofilms

**Fig 3:**
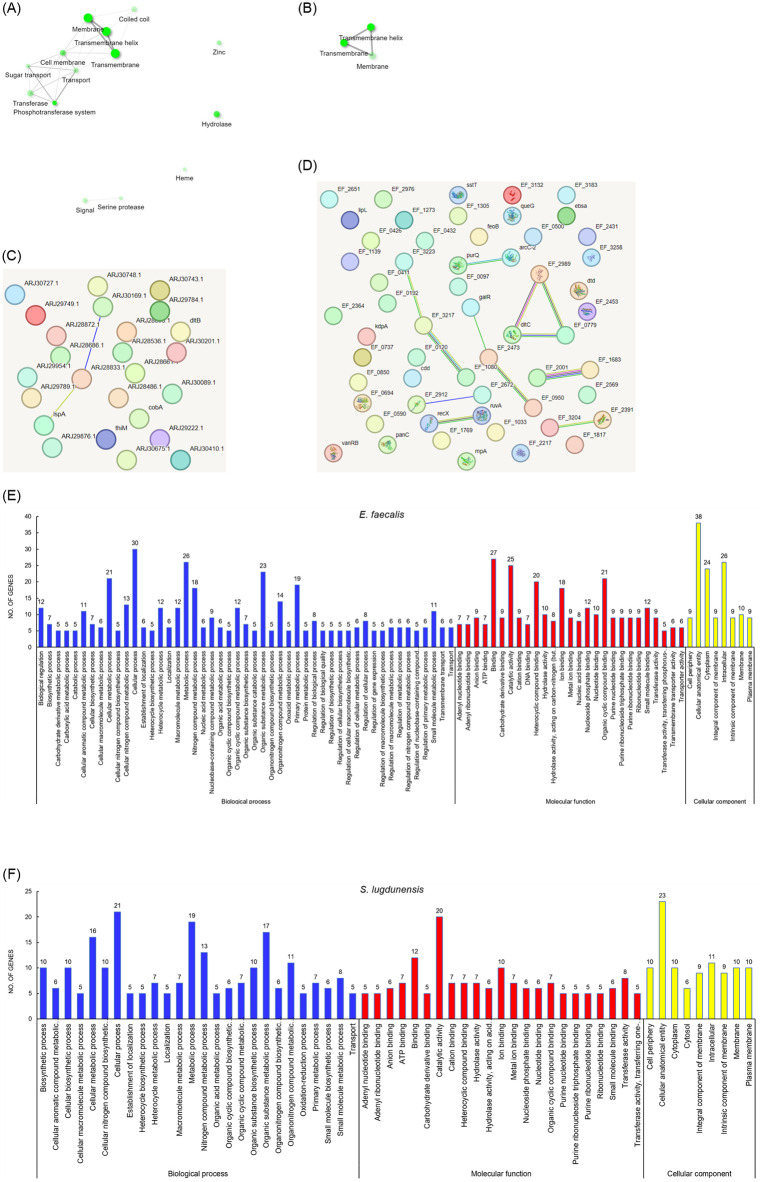
Protein network analysis and GO classification for the proteins identified only in biofilms of *E*. *faecalis* (a, c, e) and *S*. *lugdunensis* (b, d, f). **a and b.** The enrichment network of the biofilm-only proteins was generated using annotated keywords (Uniprot) as a pathway database. Darker and bigger nodes indicate more significantly enriched and larger gene sets. **c and d**. The expected protein-protein interactions obtained from STRING-db. Proteins are illustrated as nodes. Colored or white nodes indicate query proteins and the first shell of interactions, or second shell of interactions, respectively. Light blue or pink lines indicate known interactions from curated databases or experimentally determined, respectively. Some proteins are labeled with the preferred names in the website as the followings; ARJ29876.1 = EQ812_07275, ARJ29789.1 = HMPREF3225_01770, ARJ30748.1 = HMPREF3225_01013, ARJ28486.1 = HMPREF3225_00114, ARJ28895.1 = HMPREF3225_01307, ARJ30727.1 = HMPREF3225_00968, ARJ29749.1 = HMPREF3225_01812, ARJ30201.1 = EQ812_03690, ARJ28833.1 = HMPREF3225_01241, ARJ30743.1 = EQ812_05230, ARJ28872.1 = EQ812_01650, ARJ30410.1 = EQ812_09225, ARJ29784.1 = EQ812_07770, ARJ28661.1 = HMPREF3225_02342, ARJ28536.1 = EQ812_06165, ARJ30089.1 = rihC, ARJ30169.1 = HMPREF3225_00709, ARJ30675.1 = yabA, ARJ29954.1 = HMPREF3225_00598, ARJ28686.1 = HMPREF3225_02322, ARJ29222.1 = HMPREF3225_00188. **e and f**. Gene ontology (GO) classification. The differentially expressed proteins were classified into three main GO categories: biological process, molecular function, and cellular component. The number of proteins belonging to each GO term is indicated above each bar. Only GO terms with more than 5 proteins were reflected in the graph.

We analyzed the gene ontology (GO), pathways, and networks for the proteins identified only in the biofilms of each microbe (Fig 3). First, annotated keywords (Uniprot) were used as a pathway database (Table 3). Of the 59 proteins that were identified only the biofilm of *E*. *faecalis*, 23 proteins were annotated, of which 13 proteins including regulatory protein pfoR, putative (*EF_0097*), ferrichrome ABC transporter, permease protein (*EF_0192*), PTS system mannitol-specific EIICB component (*EF_0411*), polysaccharide deacetylase family protein (*EF_0590*), etc., were revealed to belong to the membrane, transmembrane or transmembrane helix, accounting for the largest portion, followed by hydrolase or transferase with 6 proteins, and cell membrane or coiled coil with 5 proteins. From the network result, it was inferred that the proteins annotated as (cell) membrane/transmembrane (helix) would be closely related to the transport system or transferase function with coiled coil structure, from which others proteins annotated as hydrolase, serine proteinase, etc. might be somehow separated. In contrast, for 53 proteins that were identified only in the biofilm of *S*. *lugdunensis*, only 9 proteins were annotated, including YycI domain-containing protein (*EQ_ EQ812_05590*), APC family permease (*EQ812_06165*), NupC/NupG family nucleoside CNT transporter (*EQ812_06755*), etc., to membrane, transmembrane or transmembrane helix. The resulting pathway networks were analyzed as depicted in Fig 3a and 3b. For both microorganisms, most annotated proteins were associated with the cell membrane, possibly being responsible for transport function.

**Table 3:** Gene annotation (GO) and pathway analysis for the proteins identified only in the biofilm of *E*. *faecalis* or *S*. *lugdunensis* using annotated keywords (Uniprot).

ID	nGenes	Pathway Genes	Fold Enrichment	Pathway	Genes
*E*. *faecalis*	13	761	3.163478853	Membrane	EF_0097 EF_0192 EF_0411 EF_0590 EF_0694 EF_0737 EF_0779 EF_1033 EF_2364 EF_2651 EF_2912 EF_3183 EF_3258
	13	735	3.275384228	Transmembrane	EF_0097 EF_0192 EF_0411 EF_0590 EF_0694 EF_0737 EF_0779 EF_1033 EF_2364 EF_2651 EF_2912 EF_3183 EF_3258
	13	735	3.275384228	Transmembrane helix	EF_0097 EF_0192 EF_0411 EF_0590 EF_0694 EF_0737 EF_0779 EF_1033 EF_2364 EF_2651 EF_2912 EF_3183 EF_3258
	6	204	5.446623094	Hydrolase	EF_0850 EF_1033 EF_1683 EF_1817 EF_2431 EF_3217
	6	323	3.43997248	Transferase	EF_0411 EF_0694 EF_1139 EF_1769 EF_2473 EF_2912
	5	168	5.511463845	Cell membrane	EF_0192 EF_0411 EF_0694 EF_2651 EF_2912
	5	292	3.170979198	Coiled coil	EF_0411 EF_0590 EF_0779 EF_1683 EF_2001
	4	159	4.658746797	Transport	EF_0192 EF_0411 EF_0694 EF_1769
	4	193	3.838034926	Signal	EF_0097 EF_1769 EF_1817 EF_3183
	3	23	24.15458937	Phosphotransferase system	EF_0411 EF_0694 EF_1769
	3	71	7.824726135	Zinc	EF_0590 EF_2431 EF_3258
	2	26	14.24501425	Sugar transport	EF_0411 EF_0694
	1	3	61.72839506	Heme	EF_1305
	1	3	61.72839506	Serine protease	EF_1817
*S*. *lugdunensis*	9	495	5.347593583	Transmembrane helix	EQ812_05590 EQ812_06165 EQ812_06755 EQ812_02465 EQ812_07275 EQ812_03435 EQ812_04050 EQ812_09000 EQ812_08585
	9	498	5.315379164	Transmembrane	EQ812_05590 EQ812_06165 EQ812_06755 EQ812_02465 EQ812_07275 EQ812_03435 EQ812_04050 EQ812_09000 EQ812_08585
	9	525	5.042016807	Membrane	EQ812_05590 EQ812_06165 EQ812_06755 EQ812_02465 EQ812_07275 EQ812_03435 EQ812_04050 EQ812_09000 EQ812_08585

Another analysis using STRING-db showed that 57 proteins out of 59 proteins from *E*. *faecalis* or 25 proteins out of 53 proteins from *S*. *lugdunensis* were annotated. The analysis results for the known or predicted protein-protein interactions indicated that the networks did not have significantly more interactions than expected (Fig 3c and 3d). However, it was clearly inferred that in *E*. *faecalis*, the interactions between phosphoribosylformylglycinamidine synthase subunit PurQ (purQ) and chorismate synthase (aroC-2), between D-alanyl carrier protein (dltC) and coenzyme A disulfide reductase (EF_2989) and glycerophosphoryl diester phosphodiesterase family protein (EF_0779), and between minor structural protein (EF_2001) and lipase/Acylhydrolase, putative (EF_1683), and in *S*. *lugdunensis* the interaction between sortase family protein (HMPREF3225_01241, “ARJ28833.1”) and putative glycolipid permease LtaA (HMPREF3225_00709, “ARJ30169.1”) would play a role in the biofilm formation or function of each microorganism.

The classification based on GO terms (biological process, molecular function, cellular component) demonstrated that “cellular process”, “catalytic activity” and “cellular anatomical entity” were highly designated in both species (Fig 3e and 3f), and in *E*. *faecalis*, “binding” was at the top rank with 27 proteins (Fig 3e). Further analysis was performed for KEGG pathways (Table 4). In both microorganisms, the two most frequently assigned common pathways were metabolic pathways (efa01100 or sln01100), followed by microbial metabolism in diverse environments (efa01120 or sln01120). However, it was indicated that there could be differences in the substances metabolized by each microorganism, e.g., fructose and mannose metabolism in *E*. *faecalis* vs. galactose metabolism in *S*. *lugdunensis*; purine metabolism in *E*. *faecalis* vs. porphyrin and chlorophyll metabolism in *S*. *lugdunensis*; arginine/proline/tryptophan metabolism in *E*. *faecalis* vs. alanine/aspartate/glutamate metabolism in *S*. *lugdunensis*.

**Table 4:** KEGG pathway analysis for the proteins identified only in the biofilm of *E*. *faecalis* or *S*. *lugdunensis*.

ID	Term ID	Term description	nGenes	Genes
*E*. *faecalis*	efa00051	Fructose and mannose metabolism	1	EF_0694
	efa00230	Purine metabolism	2	EF_2431 purL
	efa00330	Arginine and proline metabolism	1	EF_0737
	efa00380	Tryptophan metabolism	1	EF_0737
	efa00410	beta-Alanine metabolism	1	panC
	efa00450	Selenocompound metabolism	1	EF_0838
	efa00500	Starch and sucrose metabolism	1	EF_1769
	efa00564	Glycerophospholipid metabolism	1	EF_0779
	efa00627	Aminobenzoate degradation	1	EF_0737
	efa00643	Styrene degradation	1	EF_0737
	efa00770	Pantothenate and CoA biosynthesis	1	panC
	efa00785	Lipoic acid metabolism	1	lipL
	efa00790	Folate biosynthesis	1	EF_2569
	efa00970	Aminoacyl-tRNA biosynthesis	1	EF_0838
	efa01100	Metabolic pathways	7	EF_0737 EF_0694 EF_0838 EF_2431 lipL panC purl
	efa01110	Biosynthesis of secondary metabolites	2	panC purL
	efa01120	Microbial metabolism in diverse environments	2	EF_0737 EF_0694
	efa01502	Vancomycin resistance	1	vanRB
	efa02020	Two-component system	1	vanRB
	efa02060	Phosphotransferase system (PTS)	2	EF_0694 EF_1769
*S*. *lugdunensis*	sln00052	Galactose metabolism	1	EQ812_00120
	sln00250	Alanine, aspartate and glutamate metabolism	1	EQ812_04880
	sln00620	Pyruvate metabolism	1	EQ812_07770
	sln00627	Aminobenzoate degradation	1	EQ812_07770
	sln00730	Thiamine metabolism	1	thiM
	sln00790	Folate biosynthesis	1	EQ812_09225
	sln00860	Porphyrin and chlorophyll metabolism	2	EQ812_02400 cobA
	sln00910	Nitrogen metabolism	2	EQ812_04880 narH
	sln01100	Metabolic pathways	7	EQ812_02400 EQ812_07770 cobA EQ812_04880 EQ812_00120 narH thiM
	sln01110	Biosynthesis of secondary metabolites	3	EQ812_02400 cobA EQ812_04880
	sln01120	Microbial metabolism in diverse environments	5	EQ812_02400 EQ812_07770 cobA EQ812_04880 narH
	sln01230	Biosynthesis of amino acids	1	EQ812_04880
	sln01503	Cationic antimicrobial peptide (CAMP) resistance	2	dltB EQ812_09000
	sln02010	ABC transporters	1	EQ812_09000
	sln02020	Two-component system	4	EQ812_02465 dltB narH EQ812_09000
	sln02060	Phosphotransferase system (PTS)	1	EQ812_00120
	sln03060	Protein export	1	lspA
	sln04122	Sulfur relay system	1	EQ812_00500

**Fig 4:**
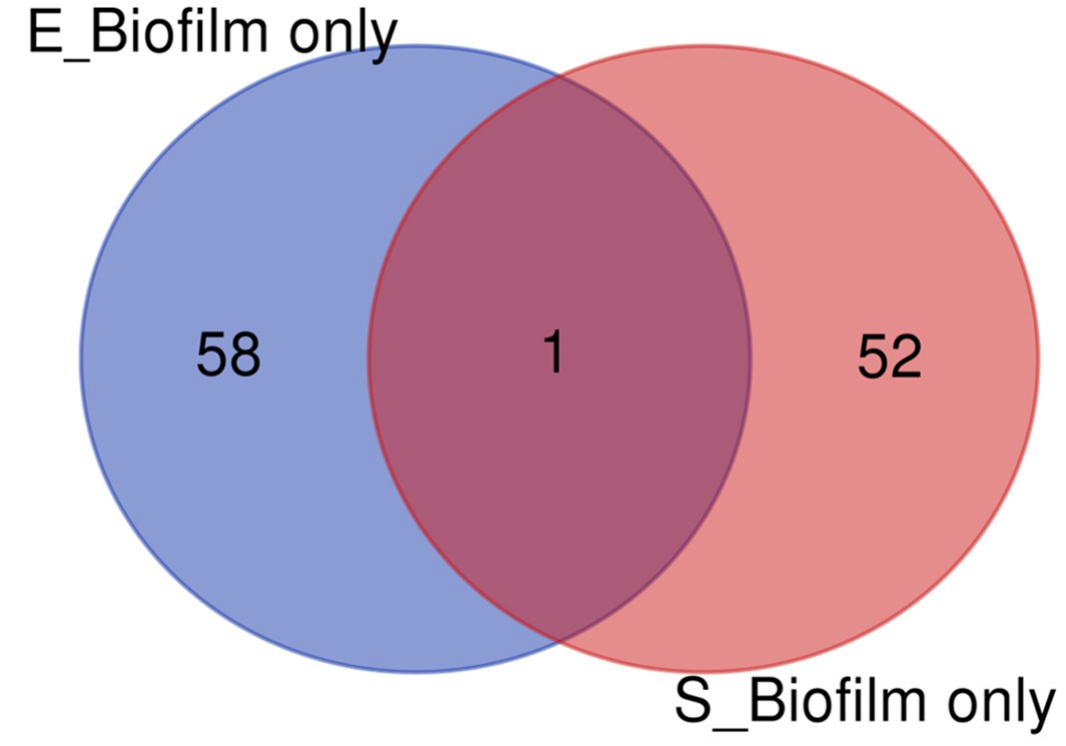
Venn diagrams for the proteins identified only in the biofilms of *E*. *faecalis* (E_Biofilm only) and *S*. *lugdunensis* (S_Biofilm only). Among the biofilm-specific 59 proteins of *E*. *faecalis* and 53 proteins of *S*. *lugdunensis*, only one protein was found in common.

Finally, we compared all the proteins identified in the biofilms of the two microbes, there were 424 proteins shared, and 505 or 701 proteins were distinguished (S3 Fig). However, when we compared the biofilm-only protein profiles of the two microbes with each other, it was discovered that only 1 protein was shared in common, which was ferrous iron transport protein B (*feoB/HMPREF3225_00188*) (Fig 4 and Table 5). This result suggested that the sets of biofilm-only proteins represented the unique feature of their contrasting abilities to form biofilms. In other words, as *E*. *faecalis* or *S*. *lugdunensis* were shown to have relatively weak or strong abilities for biofilm formation, the biofilm-specific protein sets of each microorganism might be indicators to intuitively distinguish the biofilm-forming abilities of microorganisms. Nevertheless, it was suggested that ferrous iron transport protein could be a candidate target that could commonly control the biofilm-related challenges by the two microorganisms with contrasting biofilm-forming abilities.

**Table 5:** List of proteins that were common or distinct between the biofilm-specific proteins of *E*. *faecalis* (E_Biofilm only) or *S*. *lugdunensis* (S_Biofilm only).

I.D.	nGenes	Gene name/Description
E_Biofilm only S_Biofilm only	1	Ferrous iron transport protein B
E_Biofilm only	58	ImpB/MucB/SamB family protein Heme chaperone HemW Regulatory protein pfoR, putative Lipoyl-[GcvH]:protein N-lipoyltransferase [Ribosomal protein S18]-alanine N-acetyltransferase Transcriptional regulator, AraC family Regulatory protein RecX Pyridoxal phosphate-dependent enzyme, putative CYTH domain-containing protein Cob_adeno_trans domain-containing protein Alpha-1,2-mannosidase, putative NifU family protein NusG_II domain-containing protein Lipase/Acylhydrolase, putative Ferrichrome ABC transporter, permease protein NTP_transf_3 domain-containing protein AAA domain-containing protein PC4 domain-containing protein Cobalamin synthesis protein/P47K family protein UPF0298 protein EF_2453 Ribonuclease P protein component Phosphoribosylformylglycinamidine synthase subunit PurQ Spermidine/putrescine ABC transporter, permease protein Ribosomal protein L7A family Coenzyme A disulfide reductase TraC protein Cytidine deaminase mRNA interferase HTH cro/C1-type domain-containing protein Carbamate kinase 2 Sensor histidine kinase Holliday junction ATP-dependent DNA helicase RuvA Epoxyqueuosine reductase Pantothenate synthetase Minor structural protein Cell wall surface anchor family protein, putative Protein EbsA Serine/threonine transporter SstT Surface protein PrgC Xanthine permease PTS system, fructose-specific family, IIBC components 6-aminohexanoate-cyclic-dimer hydrolase, putative Gluconate 5-dehydrogenase, putative Guanine deaminase Glutamine amidotransferase, class I Galactose operon repressor galR tRNA threonylcarbamoyladenosine biosynthesis protein TsaE Amidase, putative D-alanyl carrier protein Regulatory protein VanRB Serine protease PTS system, IIB component, putative Helicase, putative D-aminoacyl-tRNA deacylase Polysaccharide deacetylase family protein PTS system mannitol-specific EIICB component Potassium-transporting ATPase potassium-binding subunit Glycerophosphoryl diester phosphodiesterase family protein
S_Biofilm only	52	PTS system lactose-specific EIIA component TIGR00245 family protein Phosphoribosylformylglycinamidine cyclo-ligase (Fragment) 2-oxoacid:ferredoxin oxidoreductase subunit beta (Fragment) NupC/NupG family nucleoside CNT transporter Cyclase family protein (Fragment) DNA replication initiation control protein YabA Sortase family protein PTS system lactose-specific EIICB component Anthranilate synthase component I family protein Putative transcription factor FapR Solute:sodium symporter family transporter (Fragment) Cadmium efflux system accessory protein Molybdopterin biosynthesis protein MoeB Hydratase/decarboxylase Putative stage 0 sporulation protein J ATPase family protein Glutamate synthase large subunit Hydroxyethylthiazole kinase Precorrin-2 dehydrogenase MerR family transcriptional regulator Putative glycolipid permease LtaA Aquaporin family protein Putative esterase Uroporphyrinogen-III C-methyltransferase CoA-disulfide reductase Methyltransferase domain-containing protein Probable heme-iron transport system permease protein IsdF DUF5080 family protein Glyoxalase family protein Lipoprotein signal peptidase Ribonucleoside hydrolase RihC Glycosyl hydrolase family 25 Putative hemin transport system permease protein HrtB Thiamine diphosphokinase (Fragment) Choloylglycine hydrolase family protein APC family permease Acylphosphatase Metal-sulfur cluster assembly factor Teichoic acid D-alanyltransferase HAMP domain-containing histidine kinase 2-succinyl-5-enolpyruvyl-6-hydroxy-3-cyclohexene-1-carboxylate synthase (Fragment) Metallo-beta-lactamase family protein Glycosyltransferase THUMP domain protein DUF1433 domain-containing protein Nitrate reductase subunit beta DUF423 domain-containing protein Glucohydrolase (Fragment) DUF1963 domain-containing protein DUF5084 domain-containing protein YycI domain-containing protein

## Discussion

*E*. *faecalis* and *S*. *lugdunensis* are among the microorganisms that were reported to be found in situations with peri-prosthetic infections, which may cause serious complications. As peri-prosthetic infections are primarily attributed to biofilms that form on a surface, it is important to understand the biological properties of biofilm formation to properly handle the biofilm-related challenges. We previously reported that *E*. *faecalis* and *S*. *lugdunensis* exhibited contrasting biofilm-forming abilities, with the former being the weakest and the latter the strongest among the tested pathogens [12]. In this study, we examined the differences in the proteomic composition of biofilms generated under the same condition in order to gain insights for developing a strategy to treat and overcome biofilm-related infections.

When comparing the protein profiles of the biofilms and planktonic cells of the two microorganisms through the heatmap, there was no overall similarity, and the clustered genes were also found to be differentially enriched in both of the two microorganisms (Fig 1). Although the scatter plot showed up-regulated or down-regulated proteins for the proteins identified in common in biofilms and planktonic cells (Fig 2), the ratios of the abundance of biofilms to planktonic cells (B/P) were not high, which suggests that the differential expression was minimal. Therefore, we paid more attention to the proteins that were identified only in biofilms, not in planktonic cells.

For the proteins identified only in biofilms, we found that the most abundant proteins in each microbe’s biofilm were cobalamin synthesis protein/p47K family protein (EF_3204), minor structural protein (EF_2001) and regulatory protein VanRB (vanRB) in *E*. *faecalis*, or DUF5084 domain-containing protein (EQ812_04065), ATPase family protein (HMPREF3225_01770) and methyltransferase domain-containing protein (EQ812_09565) in *S*. *lugdunensis* (Table 2). Cobalamin synthesis protein/p47K family protein is known as a hydrolase that synthesizes cobalamin, in other words, vitamin B12 in *Methylomonas methanica*. Recently, cobalamin synthesis protein/p47K family protein has been reported in association with bacterial responses to antimicrobials in *E*. *faecalis* [16]. This study first presents its presence in *E*. *faecalis* biofilm, inspiring studies for the potential role of the protein in virulence by biofilm. Minor structural protein (EF_2001) was reported to be similar to *Streptococcus mitis* protein pblB derived from prophase that is in association with virulence dissemination [17]. However, the studies discovering its presence in biofilms are rare. VanRB is a DNA-binding response regulator that is related to vancomycin resistance [18], which seemed reasonable for its presence in the biofilm. DUF5084 domain-containing protein was previously reported in a genomic analysis for its involvement in the virulence of *S*. *lugdunensis* [19], but its presence in the biofilm has not been reported elsewhere. ATPase is a group of enzymes that catalyze the hydrolysis of a phosphate bond in adenosine triphosphate (ATP) to form adenosine diphosphate (ADP), one of which is known for its involvement in biofilm formation in *Staphylococcus aureus* [20]. Methyltransferase domain-containing protein may indicate a range of proteins with methyltransferase activity, which are functionally annotated in (methyl)transferase [21]. As ATPases and methyltransferase domain-containing proteins are very diverse and exist with various activities, many of them have been reported to be present in the biofilms of a variety of microorganisms, including *E*. *faecalis*, under various circumstances [22–27]. Unfortunately, among the biofilm-only proteins of each microorganism, those of the top 3 most abundant proteins mentioned above were mostly hard to match a corresponding pathway as shown in Table 3.

Nevertheless, we clearly found the obvious difference between the two groups of biofilm-only proteins, which was the presence of transport or transferase system as shown in Fig 3. Unlike the strong biofilm-forming *S*. *lugdunensis*, the weak biofilm-forming *E*. *faecalis* possessed PTS-related proteins such as PTS system mannitol-specific EIICB component (*EF_0411*), PTS system, fructose-specific family, IIBC components (*EF_0694*) and PTS system, IIB component, putative (*EF_1769*) in the biofilms. The phosphotransferase system (PTS) is a conserved active transport system in bacteria, where it catalyzes the phosphorylation of numerous carbohydrate substrates [28]. PTS has been reported in *Vibrio cholerae* and *Klebsiella pneumoniae* for its involvement in the regulation of biofilm formation [29, 30]. Especially, in *V*. *cholerae*, the role of mannitol-specific EIICB component in biofilm formation was determined through transcription of biofilm matrix exopolysaccharide synthesis genes by mannitol [31]. The relationship of the PTS system, fructose-specific IIBC component with biofilm formation was demonstrated in *Escherichia coli* [32]. Although we found that *S*. *lugdunensis* also had a PTS-related protein, it was a PTS system lactose-specific EIIA component, unlike mannitol- or fructose-specific PTS components of *E*. *faecalis*. In the aspect of a single microorganism, the finding for the presence of PTS components in biofilms was not surprising. However, considering that the two microorganisms showed contrasting abilities of biofilm formation, the role of PTS in biofilm formation according to a specific type should be determined more clearly in each microorganism.

Our proteomics results also revealed that hydrolases such as guanine deaminase (*EF_2431*) and 6-aminohexanoate-cyclic-dimer hydrolase, putative (*EF_1033*) were not only abundant but also uniquely represented in the biofilms of *E*. *faecalis*. Guanine deaminase is a hydrolytic enzyme that converts guanine to xanthine, which can affect cellular GTP and the guanylate nucleotide pool [33]. In *Chromobacterium violaceum*, the expression of guanine deaminase was controlled by CviR, which was involved in a quorum-sensing system [34]. 6-aminohexanoate-cyclic-dimer hydrolase can degrade 6-aminohexanoate-cyclic dimer, which is a xenobiotic compound [35]. The activity of 6-aminohexanoate-cyclic-dimer hydrolase was implicated in *Pseudomonas aeruginosa* for polymer degradation [36]. As the exclusive presence of guanine deaminase and 6-aminohexanoate-cyclic-dimer hydrolase in biofilm seemed to be a novel finding by this study, like PTS proteins, more studies are needed for these two proteins in order to elucidate their roles in biofilm formation of the two microorganisms utilized in this study. Nevertheless, the finding of the presence of the two enzymes in the weak biofilm-forming *E*. *faecalis* indicates the potential for usage in controlling biofilm formation.

The protein-protein interaction and network analysis shown in Fig 3 indicated that the networks did not have significantly more interactions than expected as it was essentially a random collection of proteins that were not very connected. This comment didn’t necessarily mean that it was not a biologically meaningful selection of proteins. Rather, it meant that our current set of proteins is either rather small (i.e. less than 5 proteins or so), or that it is essentially a random collection of proteins that are not very well connected, or it could simply be that these proteins have not been studied very much and that their interactions might not yet be known to STRING. This is another reason for the necessity for further studies.

The comparison of the biofilm-specific proteins of the two microorganisms revealed that ferrous iron transport protein B was solely shared in common (Fig 4). Ferrous iron transport protein is a membrane protein that is essential for Fe (II) uptake in bacteria [37]. In *E*. *faecalis*, FeoB has been reported to promote biofilm formation and antibiotic resistance by potentially binding and importing Fe2+ into the cytosol [38]. On the other hand, the presence and biological role of ferrous iron transport protein in *S*. *lugdunensis* has not yet been clearly determined, other than a previous study that found that the deletion of accessory gene regulator (agr) involved in quorum sensing upregulated ferrous iron transport protein B [39], indicating a potential involvement in biofilm formation. The function of this protein regarding biofilm regulation will be more clearly investigated through further studies.

As mentioned above, many of those proteins identified only in the biofilms were not available for GO annotation or KEGG pathway analysis, especially, the highly abundant proteins among biofilm-only proteins, and so, it was difficult to completely interpret or define our data as we originally purposed. However, this study provides new and valuable information in various aspects. First of all, there has been little proteomics data on *S*. *lugdunensis*, and information on its corresponding biofilm is even rarer. In addition, we focused on the proteins exclusively present in biofilms. The protein composition identified only in the biofilms revealed the unique set of proteins embedded only in the biofilms of each microbe, and many of those were novel for their presence or potential role in the biofilms. Therefore, this study has great significance in that it provides information on the proteomic profiling of biofilms of certain microorganisms, which is useful and helpful in identifying the exact causative bacteria and establishing customized countermeasures according to the infectious situation. Finally, although the two microorganisms with contrasting biofilm-forming abilities showed overall differences in the protein profiling of their respective biofilms, this study found that ferrous iron transport proteins were commonly present in the biofilms of both microorganisms, discovering a potential candidate target that may be able to commonly control the biofilm-related challenges by the two microorganisms.

## Supporting information

S1 Fig LC-MS chromatogram. (TIF)

S2 Fig Validation of proteomic results through RT-qPCR. **a.** Cq values of the indicated genes. **b.** Correlation analysis between Cq values obtained from RT-qPCR and abundance levels obtained from proteomics analysis.
(TIF)

S3 Fig Venn diagram for the biofilm-embedded proteins of *E*. *faecalis* and *S*. *lugdunensis*. All proteins present in their respective biofilms were compared.
(TIF)

S1 Table The proteins identified in the biofilm and planktonic cells of *E*. *faecalis*, which were a total of 971 proteins. (XLSX)

S2 Table The proteins identified in the biofilm and planktonic cells of *S*. *lugdunensis*, which were a total of 1124 proteins. (XLSX)

S3 Table A list of the top 10 proteins identified only in planktonic cells, which were not found in biofilm. (TIF)

S4 Table Primer sequences used for RT-qPCR to validate the proteomics results. (TIF)

S1 File (DOCX)
