# Supplementary figures and images for: Comparative proteomics analysis of biofilms and planktonic cells of Enterococcus faecalis and Staphylococcus lugdunensis with contrasting biofilm-forming ability
Source: PLoS One. 2024 May 29;19(5):e0298283. doi: 10.1371/journal.pone.0298283 (PMC11135667; doi:10.1371/journal.pone.0298283)

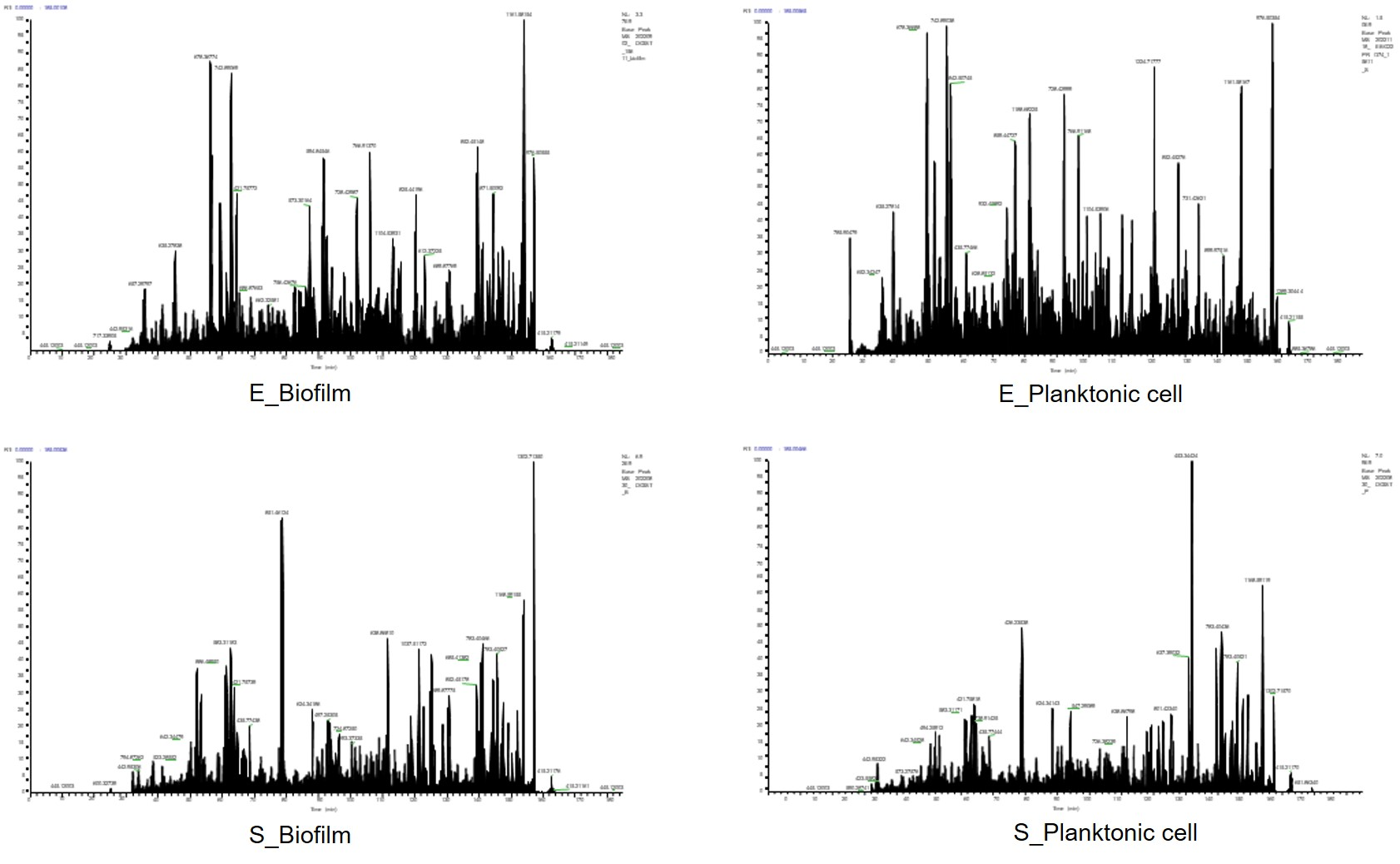

Supplement: S1 Fig — (TIF) [file pone.0298283.s001.tif]

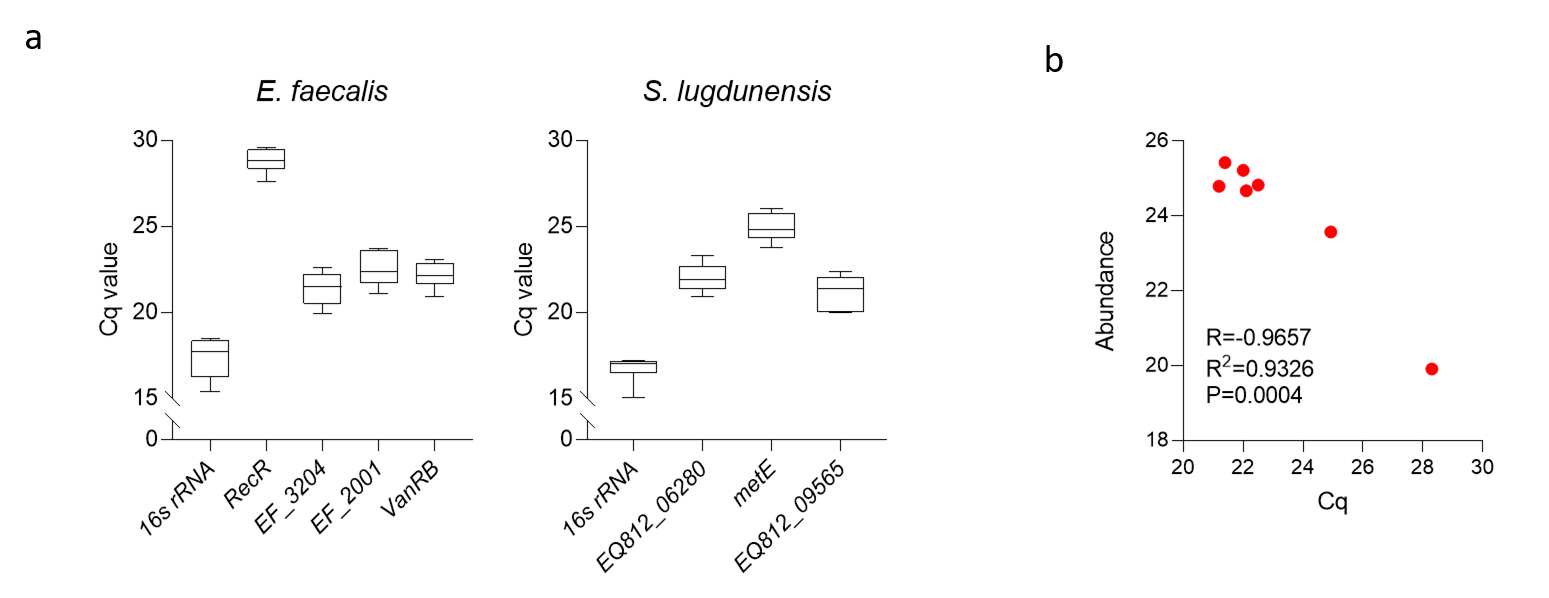

Supplement: S2 Fig — a. Cq values of the indicated genes. b. Correlation analysis between Cq values obtained from RT-qPCR and abundance levels obtained from proteomics analysis. (TIF) [file pone.0298283.s002.tif]

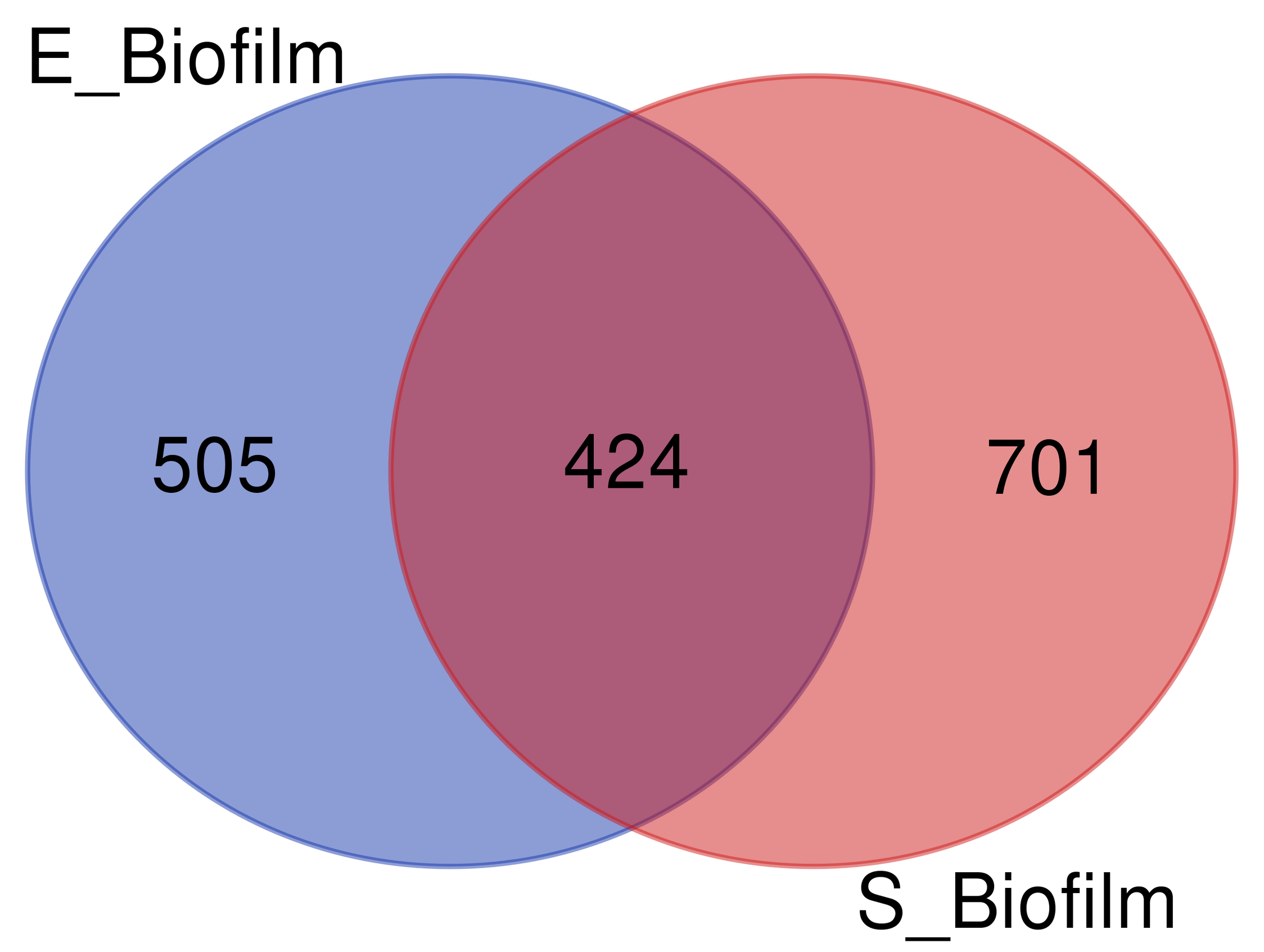

Supplement: S3 Fig — All proteins present in their respective biofilms were compared. (TIF) [file pone.0298283.s003.tif]

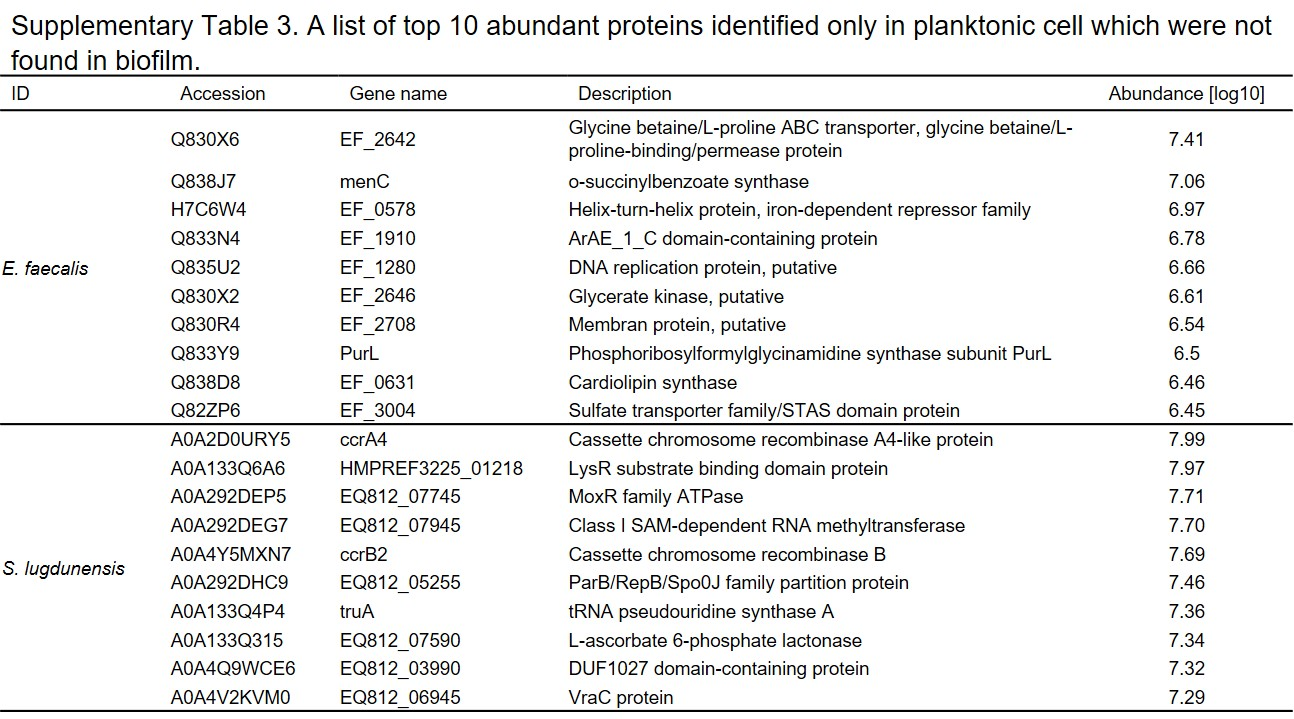

Supplement: S3 Table — (TIF) [file pone.0298283.s006.tif]

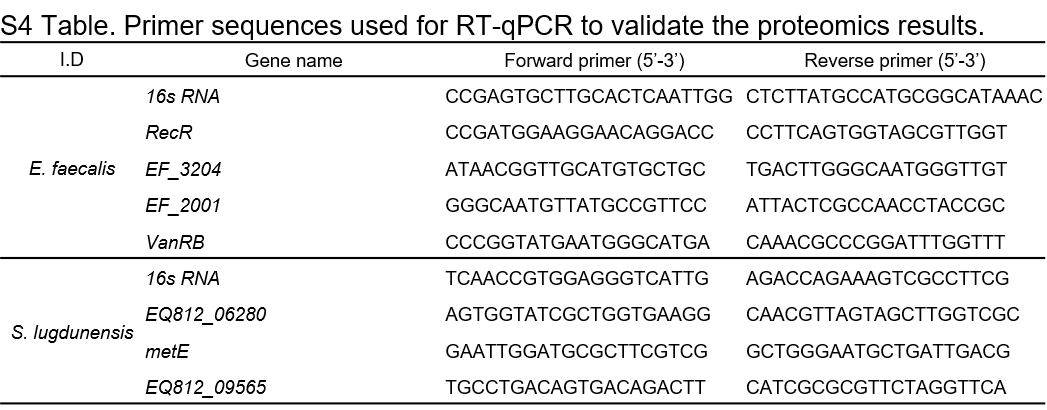

Supplement: S4 Table — (TIF) [file pone.0298283.s007.tif]
