# Supplementary material for: Comparative proteomics analysis of biofilms and planktonic cells of Enterococcus faecalis and Staphylococcus lugdunensis with contrasting biofilm-forming ability
Source: PLoS One. 2024 May 29;19(5):e0298283. doi: 10.1371/journal.pone.0298283 (PMC11135667; doi:10.1371/journal.pone.0298283)
Supplement: S1 File — (DOCX) [file pone.0298283.s008.docx]

**Comparative proteomics analysis of biofilms and planktonic cells of *Enterococcus faecalis* and *Staphylococcus lugdunensis* with contrasting biofilm-forming ability**

Jung-Ah Cho^1,2*^, Sangsoo Jeon^2^, Youngmin Kwon^1^, Yoo Jin Roh^3^, Chang-Hun Lee^3^ and Sung Jae Kim^1*^.

^1^ Department of Orthopedic Surgery, Dongtan Sacred Hospital, Hallym University, Hwaseong, Republic of Korea

^2^ College of Transdisciplinary Studies, School of Undergraduate Studies, Daegu Gyeongbuk Institute of Science and Technology, Daegu, Republic of Korea

^3^ Department of New Biology, Daegu Gyeongbuk Institute of Science and Technology, Daegu, Republic of Korea

* Corresponding author

E-mail: Jung-Ah Cho: [jungahcho@dgist.ac.kr](mailto:jungahcho@dgist.ac.kr) (JAC), Sung Jae Kim: [sung1383@hanmail.net](mailto:sung1383@hanmail.net) (SJK)

**Supplementary information**

Quantitative real-time PCR (RT-qPCR)

RNA was prepared using the Aurum Total RNA Mini Kit (BioRad). from the biofilms that had been formed on the culture tubes and harvested via vortexing with glass beads. The extracted RNA was reverse-transcribed and PCR-amplified using iScript One-Step RT-PCR Kit with SYBR Green (Bio-Rad) on Bio-Rad CFX384 real-time PCR instrument. The primers used in this study are described in S4 Table.
